# Do positive or negative experiences of social support relate to current and future health? Results from the Doetinchem Cohort Study

**DOI:** 10.1186/1471-2458-12-65

**Published:** 2012-01-21

**Authors:** Simone Croezen, H Susan J Picavet, Annemien Haveman-Nies, WM Monique Verschuren, Lisette CPGM de Groot, Pieter van't Veer

**Affiliations:** 1 Division of Human Nutrition, Academic collaborative centre AGORA, Wageningen University, Bomenweg 4, Wageningen 6703 HD, the Netherlands; 2 Department for Prevention and Health Services Research, National Institute of Public health and the Environment, Antonie van Leeuwenhoeklaan 9, Bilthoven 3721 MA, the Netherlands; 3 Academic collaborative centre AGORA, Community Health Service GGD Gelre-IJssel, Deventerstraat 43, Apeldoorn 7311 LV, the Netherlands

## Abstract

**Background:**

Cross-sectional studies have reported associations between social support and health, but prospective evidence is less conclusive. This study aims to investigate the associations of positive and negative experiences of social support with current and future lifestyle factors, biological risk factors, self-perceived health and mental health over a 10-year period.

**Methods:**

Data were from 4,724 Dutch men and women aged 26-65 years who participated in the second (1993-1997) and in the third (1998-2002) or fourth (2003-2007) study round of the Doetinchem Cohort Study. Social support was measured at round two using the Social Experiences Checklist. Health was assessed by several indicators such as smoking, alcohol consumption, physical activity, fruit and vegetable intake, overweight, hypertension, hypercholesterolemia, self-perceived health and mental health. Tertiles of positive and negative experiences of social support were analysed in association with repeated measurements of prevalence and incidence of several health indicators using generalised estimating equations (GEE).

**Results:**

Positive and negative experiences of social support were associated with prevalence and incidence of poor mental health. For the lowest tertile of positive support, odds ratios were 2.74 (95% CI 2.32-3.23) for prevalent poor mental health and 1.86 (95% CI 1.39-2.49) for incident poor mental health. For the highest tertile of negatively experienced support, odds ratios for prevalent and incident poor mental health were 3.28 (95% CI 2.78-3.87) and 1.60 (95% CI 1.21-2.12), respectively. Low levels of positive experiences of social support were also associated with low current intake of fruits and vegetables, but not with future intake. Negative experiences of social support were additionally associated with current smoking, physical inactivity, overweight and poor self-perceived health. Furthermore, high levels of negative experiences of social support were associated with future excessive alcohol consumption (OR 1.42; 95% CI 1.10-1.84), physical inactivity (95% CI 1.28; 1.03-1.58) and poor self-perceived health (OR 1.36; 95% CI 1.01-1.82).

**Conclusions:**

This study showed that social support might have a beneficial effect on lifestyle and health, with negative experiences of social support affecting lifestyle and health differently from positive experiences of social support.

## Background

A considerable amount of studies has been conducted to investigate the association between social support and health [1], including mortality [2-4], a number of chronic diseases [2,5-7], disability [8-10], cognition [11,12] and depressive symptoms and well-being [13-17]. Furthermore, social support has been found to play a role in behavioural indicators of health, such as diet and physical activity [18,19]. Although the relationship between social support and health is well established in cross-sectional studies, longitudinal evidence is less conclusive. Some studies failed to demonstrate a protective effect on changes in health, or only found a prospective association in certain subgroups [12,15]. In addition, different definitions and conceptualisations of social support have been used, making comparisons between studies difficult [20].

Behavioural pathways may mediate the relationship between social support and health [21,22]. Supportive social relationships may facilitate health promoting behaviours, such as non-smoking, non-excessive drinking, a healthy diet and physical activity. Biological processes, such as changes in cardiovascular, neuroendocrine and immune function, mediate the relationships between these behavioural processes and health. For instance, several studies demonstrated that a favourable level of social support is associated with lower blood pressure during everyday life [23,24].

Although social support is intended to be helpful, being in relationships with others can also give rise to negative experiences. Network ties may serve as potential sources of stress or can set a negative example and/or promote unhealthy behaviours. Recently, we demonstrated that positive experiences of support were related to decreased mortality over a 20-year period, whereas no effect on mortality was found for negative experiences of support [4]. This might indicate that positive and negative perceptions of support might have differential influences on health.

Little research has been carried out investigating both the positive and negative aspects of social support and their effects on health. Therefore, we explored the association between positive and negative experiences of social support and a variety of lifestyle and health indicators. We investigated influences of social support on current and future lifestyle factors, biological risk factors, poor self-perceived health and poor mental health over a period of 10 years among participants of the Doetinchem Cohort Study.

## Methods

### Design

Data were used from the second, third and fourth study round of the Doetinchem Cohort Study. The Doetinchem Cohort Study is a prospective cohort study set up to investigate the impact of lifestyle factors and biological risk factors on aspects of health in a general adult population. All participants are inhabitants of Doetinchem, a town in a rural area in the eastern part of the Netherlands, with a current population of about 56,000. More details of the Doetinchem Cohort Study are described elsewhere [25]. Ethical approval for this study was obtained by the Medical Ethical Committee TNO, Zeist, the Netherlands. During the first examination round (1987-1991) 12,405 men and women aged 20 to 59 years were examined (response rate 62%). Since then, three subsequent examination rounds have been completed in 1993-1997, 1998-2002, and 2003-2007. For round 2, a random two-third of those who were measured in round one were re-invited; 3,641 men and 4,128 women aged 26-65 years. For round 3 and 4, people who participated in the previous study round were invited, excluding those who emigrated, actively withdrew from the study or had died. Response rates were 79% for round 2, 75% for round 3, and 78% for round 4. Participants received a general and a dietary questionnaire to complete at home, and were invited to attend a medical examination at the community health service, where blood samples were collected, blood pressure was measured, and anthropometric measurements were taken. During the first study round, a number of variables of interest for present study were not collected. Therefore, round 2 will be considered the baseline measurement for present analyses.

### Measurements

#### Social support

Perceived social support was measured at round 2 by the Social Experiences Checklist [26,27]. The Social Experiences Checklist reflects experiences in social relationships and consists of 16 items, of which eight items correspond to positive experiences of social support and eight items correspond to negative experiences of social support. The instrument was validated among middle-aged Dutch adults by Van Oostrom et al. (1995), who tested and confirmed two hypotheses: 1) a negative correlation of neuroticism with positive experiences of social support and a positive correlation with negative experiences of social support, and 2) a positive correlation between positively experienced social support and active coping [27]. The reliability of the Social Experiences Checklist was found to be moderate over a six-year period: test-retest correlation coefficient of 0.49 for both positive and negative experiences of support [4]. Positive experiences included items such as warmth and friendliness, esteem, and help. An example of these positively formulated experiences is: "How often did you experience in your contacts with other people warmth and friendliness?" Negative experiences included items such as lack of understanding, belittlement, and avoidance, e.g.: "How often did you experience in your contacts with other people that someone belittled you?" Responses to these questions were formulated on a 4-point Likert-type scale, with response categories: "never", "sometimes", "regularly", or "often". Levels of positive and negative experiences of social support were determined by creating tertiles of the sum scores [4].

#### Socio-demographic characteristics

At round 2, sex, age, ethnicity, marital status, educational level, and employment status were collected using a general questionnaire. Marital status was assessed as being married or not married. Educational level was divided into three categories: low (primary school, lower vocational education or less), medium (medium vocational education, higher secondary level education) and high (higher vocational education, university). Employment status was dichotomised into having a paid job, yes or no.

#### Lifestyle factors

Smoking, alcohol consumption, fruit and vegetable intake and physical activity were assessed by means of a questionnaire at round 2, 3 and 4. Smoking was categorised into current, former and never smoking. Alcohol consumption was dichotomised into drinking less than 2 glasses a day and drinking at least 2 glasses a day. Total fruit and vegetable intake was assessed using a validated semi-quantitative food-frequency questionnaire [28]. Having a total fruit and vegetable intake less than median intake at round 2 was categorised as "low". Physical activity was repeatedly measured with identical questionnaires from 1994 onwards (i.e. the second year of round 2). Data on physical activity were collected by use of a questionnaire on physical activity developed for the EPIC study, extended with questions on sports and other strenuous leisure-time activities [29]. This questionnaire, without the later added item on sports, was validated with a 3-day activity diary among people aged 20-70 years [29]. The authors demonstrated satisfactory reproducibility and relative validity of the ranking of subjects. The questionnaire included questions on time spent walking, cycling, doing odd jobs, gardening, asked for summer and winter separately, and sports and occupational activities irrespective of season. To determine the activity levels of the participants, the total time (hours/week) spent on physical activities was calculated. Being physically inactive was defined as being active with moderate intensity (4 to 6.5 MET) for less than 3.5 hours per week.

#### Biological risk factors

At each round, biological risk factors were measured. Weight and height were measured to the nearest 0.1 kg and 0.5 cm, respectively. Participants were weighed wearing indoor clothing, without shoes and with empty pockets. Accordingly, 1 kg was subtracted from the measured weights to take the clothing into account. Based on calculated body mass index (kg/m^2^) respondents were allocated to either having a normal weight (< 25 kg/m^2^) or having overweight (≥ 25 kg/m^2^) [30]. Blood pressure was measured twice with participants in sitting position, according to a standardised procedure. Hypertension was defined as having a mean systolic blood pressure of ≥ 140 mm Hg and/or diastolic blood pressure of ≥ 90 mm Hg and/or use of anti-hypertensive drugs [31]. Cholesterol values were determined in non-fasting blood serum, which was stored at -20°C for a maximum of three weeks. Hypercholesterolemia was defined as having a total cholesterol level of ≥ 6.5 mmol/L and/or use of cholesterol-lowering medication.

#### Self-perceived health and mental health

Self-perceived health and mental health were assessed from 1995 onwards (i.e. the third year of round 2). Self-perceived health was ascertained by asking: "how would you rate your health in general?", using a five-point scale ranging from poor to excellent. Poor self-perceived health was defined as having moderate or poor health. The Mental Health Index (MHI-5) was used to measure general mental health. The index is widely used, has a good reliability and it performs comparable to its longer MHI counterparts [32,33]. It consists of the following five questions "How much of the time in the previous four weeks: have you been a very nervous person?, have you felt so down in the dumps that nothing could cheer you up?, have you felt calm and peaceful?, have you felt downhearted and blue?, have you been a happy person?". The six response categories (all, most, often, some, a little, or none of the time) were transformed into standardised MHI-5 scores ranging from 0 (poor mental health) to 100 (excellent mental health). Poor mental health was determined as having a score of below 61 [34-36].

### Data analysis

Participants with complete data on all positive or negative items of the Social Experiences Checklist (n = 4,714; 94.3% for the positive items, and n = 4,741; 94.9% for the negative items), and the socio-demographic variables (n = 4,860; 97.2%) were included in the analysis, resulting in an analytical sample of 4,724 participants. We used Generalised Estimating Equations (GEE) to estimate population-averaged associations between social support and 1) the prevalence and 2) the incidence of different lifestyle and health indicators over 10 years of follow-up. The GEE analyses were carried out with an exchangeable correlation structure.

First, we analysed if positive and negative experiences of social support, measured at round 2, were associated with the occurrence of unhealthy lifestyle factors, biological risk factors, poor self-perceived health and poor mental health measured at round 2, 3 and 4. The advantage of using GEE over traditional cross-sectional logistic regression analysis here is that GEE incorporates all repeated measurements of the health outcomes and accounts for the dependency between them [37]. Second, we explored the role of social support on changes in our health measures by investigating if positive and negative experiences of social support, measured at round 2, were related to the incidence of unhealthy lifestyle, biological risk factors, poor self-perceived health and poor mental health, measured at rounds 3 and 4. To analyse this association between social support and incident health outcomes, a subgroup of participants was used who did not have the health outcome at round 2.

All GEE models included the socio-demographic characteristics sex, age, educational level and marital status as confounding factors. Time was represented in the models by the categorical variable "study round". Sex was considered an effect modifier, but interaction terms with positive and negative experiences of social support were not statistically significant (*p *< 0.05). Ethnicity was not included in the models, since only 1.1% of the participants were from a non-Dutch origin. In addition, when analysing one of the unhealthy lifestyle factors, models were additionally adjusted for the other lifestyle factors. In the same manner, the models for one of the biological factors were additionally adjusted for the other biological factors, and models for self-perceived health were additionally adjusted for mental health and vice versa. Statistical analyses of the data were carried out using version 9.1 of the SAS software program. In all analyses, *P*-values < 0.05 were considered to be statistically significant.

## Results

The mean age of the study population at round 2 was 45 years (standard deviation (SD): 10). Further characteristics of the participants as well as baseline prevalence of lifestyle characteristics, biological risk factors and health indicators are described in Table 1. About half of the participants were female (52%), 82% were married and 64% were employed. One fifth of the participants were higher educated. This group perceived higher levels of positive support than the lower educated participants did. Concerning lifestyle characteristics, unfavourable levels of fruit and vegetable intake were highest in participants who experienced the lowest levels of positive support (55%). Although the prevalence of smoking and overweight considerably increased over rising levels of negative experiences of social support, the number of hypertensive participants was lowest among those experiencing high levels of negative support (23%). Poor self-perceived health and poor mental health were most profound for low levels of positive support and high levels of negative support.

**Table 1:** General, lifestyle and health characteristics by tertiles of positive and negative experiences of social support

	Positive experiences of social support (n = 4,592)			Negative experiences of social support (n = 4,616)		
	**T3 (high)**	**T2**	**T1 (low)**	**T1 (low)**	**T2**	**T3 (high)**
	**(25-32)**	**(22-24)**	**(8-21)**	**(8-11)**	**(12-13)**	**(14-32)**

*Sex (%)*						

Men	43.1	48.8	52.6	43.6	47.8	52.7

Women	56.9	51.2	47.4	56.4	52.2	47.3

Age, years (mean, SD)	44.2 (10.0)	45.4 (10.1)	46.4 (9.6)	46.3 (10.3)	45.1 (9.9)	44.7 (9.6)

*Marital status (%)*						

Married	80.1	84.0	81.9	83.2	83.2	79.8

Unmarried	19.9	16.0	18.1	16.8	16.8	20.2

*Educational level (%)*						

Low	48.8	50.2	58.5	55.1	48.6	52.8

Medium	28.2	28.1	25.4	24.0	29.7	28.2

High	23.0	21.7	16.1	20.8	21.7	19.0

*Employment status (%)*						

Employed	66.8	65.8	61.6	58.9	66.7	68.4

Unemployed	33.2	34.2	38.4	41.1	33.3	31.6

*Lifestyle characteristics (%)*						

Current smoking	30.8	28.5	29.5	25.7	28.9	34.2

Excessive alcohol consumption	22.2	22.0	22.6	20.7	23.3	23.1

Low fruit and vegetable intake	44.1	50.4	55.1	49.3	48.7	52.1

Physical inactivity	21.4	20.5	23.1	20.1	21.3	23.6

*Biological factors (%)*						

Overweight	48.4	50.4	51.6	47.8	48.7	52.9

Hypertension	22.8	26.2	25.6	26.5	25.3	22.7

Hypercholesterolemia	15.2	14.6	16.4	15.2	15.1	15.6

*Health indicators (%)*						

Poor self-perceived health	7.5	10.5	13.0	5.4	8.2	15.9

Poor mental health	9.2	13.3	27.5	6.4	12.6	27.9

*N*: Number of participants; *SD*: Standard deviation

Associations between experiences of social support and the prevalence and incidence of different health indicators over 10 years of follow-up are presented in Tables 2 and 3. Results show a consistent effect of both positive and negative experiences of support on the prevalence and incidence of poor mental health. For low levels of positive support, the odds ratio (OR) for prevalence was 2.74 (95% confidence interval (CI) 2.32-3.23) and the OR for incidence was 1.86 (95% CI 1.39-2.49). For high levels of negative experiences of support, the ORs for prevalent and incident poor mental health were 3.28 (95% CI 2.78-3.87) and 1.60 (95% CI 1.21-2.12), respectively. Low levels of positive experiences of social support were further associated with the prevalence of low fruit and vegetable intake (OR 1.36; 95% CI 1.20-1.53), but not with the incidence. Negative experiences of social support on the other hand, were associated with four other prevalent outcomes besides mental health. High levels of negative experiences of support were associated with prevalent smoking (OR 1.39; 95% CI 1.20-1.61), physical inactivity (OR 1.23; 95% CI 1.08-1.41), overweight (OR 1.23; 95% CI 1.09-1.40) and poor self-perceived health (OR 2.17; 95% CI 1.81-2.60). The association between negative experiences of social support and prevalent overweight was independent of lifestyle (data not shown). Furthermore, high levels of negative experiences of social support were additionally associated with the incidence of excessive alcohol consumption (OR 1.42; 95% CI 1.10-1.84), physical inactivity (OR 1.28; 95% CI 1.03-1.58) and poor self-perceived health (OR 1.36; 95% CI 1.01-1.82). Neither positive nor negative experiences of social support were associated with hypertension or hypercholesterolemia.

**Table 2:** Experiences of social support and prevalent lifestyle characteristics, biological factors, self-perceived health and mental health

	Positive experiences of social support					Negative experiences of social support				
	**T3 (high)**	**T2**		**T1 (low)**		**T1 (low)**	**T2**		**T3 (high)**	

	**OR**	**OR**	**95% CI**	**OR**	**95% CI**	**OR**	**OR**	**95% CI**	**OR**	**95% CI**

*Lifestyle characteristics^1^*										

Current smoking	1	0.92	0.79-1.07	1.00	0.86-1.16	1	1.18	1.01-1.39	1.39	1.20-1.61

Excessive alcohol consumption	1	0.93	0.80-1.09	0.92	0.78-1.08	1	1.07	0.91-1.27	1.06	0.91-1.24

Low fruit and vegetable intake	1	1.28	1.14-1.45	1.36	1.20-1.53	1	0.94	0.83-1.06	1.00	0.89-1.12

Physical inactivity	1	0.92	0.81-1.06	1.00	0.87-1.14	1	1.18	1.03-1.36	1.23	1.08-1.41

*Biological factors^2^*										

Overweight	1	0.96	0.84-1.09	0.93	0.81-1.06	1	1.04	0.90-1.19	1.23	1.09-1.40

Hypertension	1	1.07	0.94-1.22	1.00	0.88-1.14	1	0.97	0.85-1.11	0.95	0.84-1.08

Hypercholesterolemia	1	0.90	0.77-1.04	1.03	0.89-1.19	1	1.04	0.90-1.21	1.06	0.92-1.22

*Health indicators^3^*										

Poor self-perceived health	1	1.04	0.87-1.25	1.13	0.94-1.35	1	1.49	1.21-1.82	2.17	1.81-2.60

Poor mental health	1	1.30	1.09-1.55	2.74	2.32-3.23	1	1.49	1.23-1.80	3.28	2.78-3.87

*CI*: confidence interval; *OR*: odds ratio; *T: *tertile

^1 ^models included sex, age, educational level, marital status, employment status, study round, smoking, alcohol consumption, fruit and vegetable intake and physical activity

^2 ^models included sex, age, educational level, marital status, employment status, study round, overweight, hypertension and hypercholesterolemia

^3 ^models included sex, age, educational level, marital status, employment status, study round, self-perceived health and mental health

**Table 3:** Experiences of social support and incident lifestyle characteristics, biological factors, self-perceived health and mental health

	Positive experiences of social support					Negative experiences of social support				
	**T3 (high)**	**T2**		**T1 (low)**		**T1 (low)**	**T2**		**T3 (high)**	

	**OR**	**OR**	**95% CI**	**OR**	**95% CI**	**OR**	**OR**	**95% CI**	**OR**	**95% CI**

*Lifestyle characteristics^1^*										

Current smoking	1	1.11	0.73-1.68	1.31	0.86-2.00	1	1.24	0.83-1.87	1.08	0.72-1.60

Excessive alcohol consumption	1	1.04	0.79-1.35	0.99	0.75-1.31	1	1.21	0.91-1.62	1.42	1.10-1.84

Low fruit and vegetable intake	1	1.16	0.95-1.41	1.03	0.84-1.28	1	0.98	0.79-1.21	1.08	0.89-1.32

Physical inactivity	1	0.89	0.71-1.10	1.05	0.84-1.29	1	1.39	1.12-1.73	1.28	1.03-1.58

*Biological factors^2^*										

Overweight	1	0.97	0.78-1.19	1.05	0.85-1.29	1	0.82	0.66-1.01	1.05	0.86-1.27

Hypertension	1	1.04	0.89-1.22	1.08	0.92-1.27	1	0.98	0.83-1.15	1.05	0.90-1.22

Hypercholesterolemia	1	0.96	0.79-1.16	1.07	0.88-1.29	1	0.98	0.81-1.19	0.98	0.82-1.18

*Health indicators^3^*										

Poor self-perceived health	1	0.95	0.71-1.28	0.81	0.59-1.11	1	1.05	0.76-1.45	1.36	1.01-1.82

Poor mental health	1	1.17	0.87-1.58	1.86	1.39-2.49	1	0.94	0.69-1.28	1.60	1.21-2.12

*CI*: confidence interval; *OR*: odds ratio; *T*: tertile

^1 ^models included sex, age, educational level, marital status, employment status, study round, smoking, alcohol consumption, fruit and vegetable intake and physical activity

^2 ^models included sex, age, educational level, marital status, employment status, study round, overweight, hypertension and hypercholesterolemia

^3 ^models included sex, age, educational level, marital status, employment status, study round, self-perceived health and mental health

## Discussion

This study showed that unfavourable levels of positive and negative experiences of social support were associated with poor mental health and with changes from a good to a poor mental health. Low levels of positive experiences of social support were additionally associated with a low fruit and vegetable intake. High levels of negative experiences of social support on the other hand, were additionally associated with smoking, physical inactivity, overweight and poor self-perceived health, and with unhealthy changes in alcohol consumption, physical activity and self-perceived health. Neither positive nor negative experiences of social support were associated with hypertension or hypercholesterolemia.

Although the relationship between social support and mental health is already well established in existing literature, results regarding other lifestyle and health outcomes are however inconclusive [14]. Investigating the unhealthy changes over a 10-year period enabled us to estimate the associations between social support and the onset of different lifestyle and health indicators. This analytical strategy was also applied by a few other studies [12,15,18,38]. Glass et al. (2006) reported that social engagement, defined as social and productive activity, was associated with prevalent and incident depressive symptoms, whereas Melchior et al. (2003) found a significant association between inadequate social support and incident self-reported poor health in men, but not in women [15,38]. Green et al. (2008) only found significant associations between social network characteristics and cognitive and functional status but not decline [12]. The researchers suggested that cognitive and functional decline might therefore be the cause rather than the consequence of social network characteristics [12]. This might also be partly explaining why we have found an association between social support and current smoking and fruit and vegetable intake, but not for future smoking or intake of fruits and vegetables. Regarding physical activity, our results showed that negative experiences of social support related to current and future physical inactivity. This corroborated with the findings of Kouvonen et al. (2011) that practical support was associated with the recommended amount of leisure time physical activity in cross-sectional and longitudinal analysis. However, they did not find a longitudinal association concerning confiding/emotional support and incident physical activity [18]. In our study we used positive and negative experiences of social support as separate predictors of health. This led to the result that smoking, physical inactivity, overweight and self-perceived health appeared to be more affected by negative experiences of support than by positive experiences of support. Newsom et al. (2005), who studied the relative importance of positive and negative exchanges, found that positive exchanges only related to well-being, whereas negative exchanges additionally related to psychological distress [39]. This could indicate that different mechanisms for positive and negative experiences of support may play a role in how they influence health.

### Strengths and limitations

We studied the relationship between social support and a variety of health outcomes. Outcomes with different levels of subjectivity were included ranging from subjective outcomes (mental health and self-perceived health) to less subjective outcomes (lifestyle factors) to objective outcomes (clinically measured factors), in order to overcome the problem of correlated measurement error. Correlated measurement error is likely to occur when both the exposure and the outcome measurement have the same source of error [40]. This is particularly of concern when both measurements have high levels of subjectivity, like with social support and self-reported health. Furthermore, we tried to diminish the bias caused by correlated measurement error by using repeated measurements to estimate the prevalence of the outcomes. Additionally, we studied the association with incident outcomes, hereby excluding the prevalent cases of poor health at round 2. Nevertheless, correlated measurement error still could be a methodological explanation for both the found association between social support and self-perceived health or mental health, and the lack of association between social support and hypertension or hypercholesterolemia. Also, correlated measurement error could have played a role in the association between 1) positive experiences of social support and low fruit and vegetable intake, and 2) negative experiences of social support and current smoking. Concerning both outcomes, a statistically significant association was found for the prevalent outcomes but not the incident outcomes. Another limitation of this study was that although we used a prospective analytical approach, we cannot provide evidence that improving the quality of social support leads to a more beneficial lifestyle and improves health status. This is because we had no information about changes in social support prior to our measurement, and were therefore not able to investigate the effects of changes in social support on lifestyle and health.

Another strength of our study was the low number of non-response regarding the Social Experiences Checklist. Participants were excluded if they had one or more missing items on the Social Experiences Checklist. Because each questionnaire was checked for item non-response at the community health service by a trained research assistant, the number of people with missing items for both the positive and negative experiences of social support was low (2.9%). People with missing values for the social support assessment were likely to be older, lower educated and/or more often unemployed than the analytical sample. However, the influence of age, educational level and employment status on the risk estimates was not larger than the effects of social support on the different health indicators. Therefore, we believe that any attenuation of the effects of social support on the different health outcomes caused by this selection bias is minimal.

### External validity

Social support as determinant of health is complex and closely interwoven with other factors, including socio-economic and cultural factors [41,42]. The participants included in our study lived in a rural area and almost all of them originated from the Netherlands. Research in other populations and countries is needed to gain insight in the universal impact of social support on health and health-related factors. For the analysis in this study, the second, third and fourth round of the Doetinchem Cohort Study were used. Although the response rates were generally good throughout these three subsequent rounds (79%, 75% and 78% respectively), we cannot rule out the possibility that attrition due to drop-outs or intermittent missing data in the outcome variables might have influenced our results. Nevertheless, this possible selection bias would mainly affect the prevalence estimates, and much less the estimated magnitudes of the associations [25].

### Implications

The results of this study imply that both positive and negative aspects of social support are related to some, but not all, indicators of lifestyle and health. More research that differentiates between positive and negative experiences of social support is necessary to confirm our findings, and to give insight into how they may operate differently on health. In our analysis, we assessed social support at a single time point. Longitudinal studies using repeated measurements of social support are needed to investigate the influence of changes in social support on lifestyle and health, before any recommendations regarding public health interventions can be given.

## Conclusions

To conclude, favourable experiences of social support may have a beneficial effect on lifestyle and health. Additional longitudinal studies addressing positive and negative experiences of social support are needed to replicate our finding. Conclusive evidence of the associations between positive and negative experiences of social support and lifestyle and health would enhance tailoring future intervention strategies to promote a healthy lifestyle and improve health.

## Competing interests

The authors declare that they have no competing interests.

## Authors' contributions

WMMV supervised the acquisition of data. SC, HSJP, AH, LCPGMG and PV designed the study's analytic strategy. SC conducted the data analysis and drafted the article. All authors contributed to the interpretation of the data, and reviewed and revised the article. All authors read and approved the final manuscript.

## Pre-publication history

The pre-publication history for this paper can be accessed here:

http://www.biomedcentral.com/1471-2458/12/65/prepub
